# Diabetes as a risk factor for heart failure in women and men: a systematic review and meta-analysis of 47 cohorts including 12 million individuals

**DOI:** 10.1007/s00125-019-4926-x

**Published:** 2019-07-18

**Authors:** Toshiaki Ohkuma, Yuji Komorita, Sanne A. E. Peters, Mark Woodward

**Affiliations:** 1 The George Institute for Global Health, University of New South Wales, Level 10, King George V Building, Royal Prince Alfred Hospital, Missenden Road, Camperdown, Sydney, NSW 2050 Australia; 2 Department of Medicine and Clinical Science, Graduate School of Medical Sciences, Kyushu University, Fukuoka, Japan; 3 Division of Internal Medicine, Fukuoka Dental College, Fukuoka, Japan; 4 The George Institute for Global Health, University of Oxford, Hayes House, 75 George Street, Oxford, OX1 2BQ UK; 5 Department of Epidemiology, Johns Hopkins University, Baltimore, MD USA

**Keywords:** Diabetes, Heart failure, Meta-analysis, Sex differences, Systematic review

## Abstract

**Aims/hypothesis:**

The prevalence of diabetes and heart failure is increasing, and diabetes has been associated with an increased risk of heart failure. However, whether diabetes confers the same excess risk of heart failure in women and men is unknown. The aim of this study was to conduct a comprehensive systematic review with meta-analysis of possible sex differences in the excess risk of heart failure consequent to diabetes. Our null hypothesis was that there is no such sex difference.

**Methods:**

A systematic search was conducted in PubMed for population-based cohort studies published between January 1966 and November 2018. Studies were selected if they reported sex-specific estimates of RRs for heart failure associated with diabetes, and its associated variability, which were adjusted at least for age. Random-effects meta-analyses with inverse variance weighting were used to obtain pooled sex-specific RRs and women-to-men ratio of RRs (RRRs) for heart failure associated with diabetes.

**Results:**

Data from 47 cohorts, involving 12,142,998 individuals and 253,260 heart failure events, were included. The pooled multiple-adjusted RR for heart failure associated with type 1 diabetes was 5.15 (95% CI 3.43, 7.74) in women and 3.47 (2.57, 4.69) in men, leading to an RRR of 1.47 (1.44, 1.90). Corresponding pooled RRs for heart failure associated with type 2 diabetes were 1.95 (1.70, 2.22) in women and 1.74 (1.55, 1.95) in men, with a pooled RRR of 1.09 (1.05, 1.13).

**Conclusions/interpretation:**

The excess risk of heart failure associated with diabetes is significantly greater in women with diabetes than in men with diabetes.

PROSPERO registration: CRD42019135246

**Electronic supplementary material:**

The online version of this article (10.1007/s00125-019-4926-x) contains peer-reviewed but unedited supplementary material, which is available to authorised users.

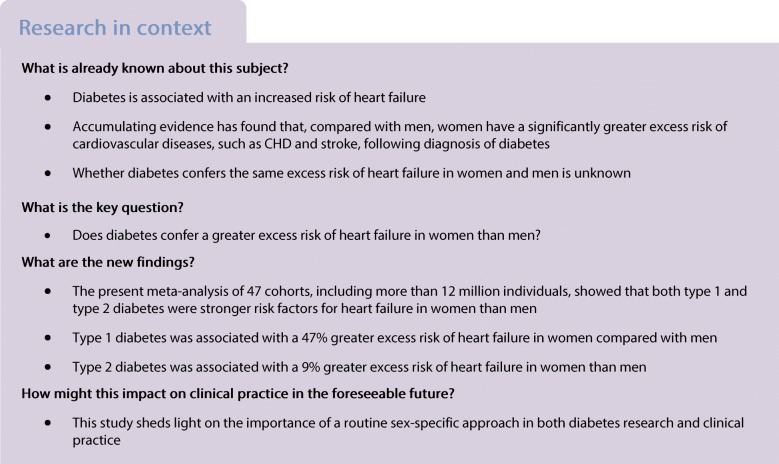



## Introduction

Diabetes and heart failure are now recognised as frequent comorbid conditions; the prevalence of type 2 diabetes in individuals with heart failure was reported to be 4.3–28%, whilst that of heart failure in those with type 2 diabetes was reported to be 12–57% [1]. Diabetes is associated with an increased risk of heart failure [2], and also increases the risk of premature death after diagnosis of heart failure [3, 4]. Furthermore, heart failure is the second most common initial presentation of cardiovascular disease in people with type 2 diabetes and more common than myocardial infarction or stroke [5]. Although heart failure appears to be a complication of diabetes [6], this is still not fully recognised [1]. The number of people with heart failure is expected to increase continuously in the future, and thus efficient earlier prevention and treatment of heart failure is crucial.

Accumulating evidence has found that there are considerable sex differences in the excess risk of cardiovascular diseases associated with diabetes [7]. Our previous meta-analyses have shown that, compared with men, women have a significantly greater excess risk of CHD [8], stroke [9], as well as the non-cardiovascular complications of dementia [10], and cancer [11], following diabetes. However, whether these associations are also observed for heart failure is unknown, as the previous meta-analysis on the diabetes–heart failure association [2] included single-sex studies, which may have led to unreliable results due to differences in methodology, confounding factors included and background risk between the studies of women alone and men alone. Herein, we report the most comprehensive systematic review of the literature with a meta-analysis of possible sex differences in the excess risk of heart failure consequent to diabetes using only studies that included both sexes.

## Methods

### Search strategy and selection criteria

We conducted a systematic search in PubMed on 16 November 2018 using a combination of text words and medical subject headings (electronic supplementary material [ESM] Table 1). The reference lists of identified studies were also reviewed to identify other relevant studies.

Observational cohort studies were included if they had provided sex-specific RRs, or equivalents, for the association between diabetes and heart failure in both women and men. Studies were excluded if they were cohorts based on individuals with any underlying diseases, reported data for a single sex only, did not adjust at least for age, or did not provide information about the variability around the point estimate. In cases of duplicate reports from the same study, the study providing the longest follow-up or the highest number of events was included. Two authors (T. Ohkuma and Y. Komorita) conducted the search and extracted the data independently, and uncertainties regarding the inclusion of studies and data extraction were discussed and resolved by mutual consent. The meta-analysis was conducted in accordance with Meta-analysis Of Observational Studies in Epidemiology (MOOSE) guidelines [12].

### Data extraction and statistical analysis

The primary outcome was incident heart failure (either fatal or non-fatal). The primary metrics were the pooled multiple-adjusted sex-specific RRs and the women-to-men ratio of RRs (RRRs) for heart failure, comparing individuals with diabetes with those without diabetes. In pooling multiple-adjusted RRs, the set of adjustments made was allowed to vary by study, but had to include at least one other risk factor for heart failure, in addition to age. Multiple RRs of subgroups from one study were combined into a single RR using a fixed-effect model. The pooled estimates of sex-specific RRs across studies were computed using random-effects meta-analyses with inverse variance weighting applied on the log scale. The same method was used to pool the RRRs. Data on type 1 and type 2 were separately pooled, where studies which did not differentiate type of diabetes were classified as type 2, which accounts for about 90–95% of all individuals with diabetes [13]. The *I*^2^ statistic was used to estimate the percentage of variability across studies due to between-study heterogeneity. Cochran’s *Q* test was used to assess whether there was a significant between-study heterogeneity.

Age-adjusted RRs were also pooled separately in secondary analyses. A sensitivity analysis was conducted to compare multiple-adjusted and age-adjusted estimates, where the studies were restricted to those that reported both. The presence of publication bias was examined using funnel plots and Egger’s and Begg’s tests. Meta-regression analyses tested for differences between prespecified subgroups in multiple-adjusted analyses: study region (Asia or non-Asia), year of baseline study (pre-1985 or 1986 onwards), ascertainment of diabetes (self-reported only or others), study outcome (fatal only or fatal and non-fatal combined), study quality (the Newcastle-Ottawa Scale [14] [ESM text and ESM Table 2], ≥8 or <8 points) and by absolute risk differences (greater in men or greater in women). Since only two studies were identified for type 1 diabetes, the analyses described in this paragraph were only applied for type 2 diabetes.

A *p* value <0.05 was considered to be statistically significant. All analyses were performed using Stata software (release 13; StataCorp, College Station, TX, USA).

## Results

**Fig. 1:**
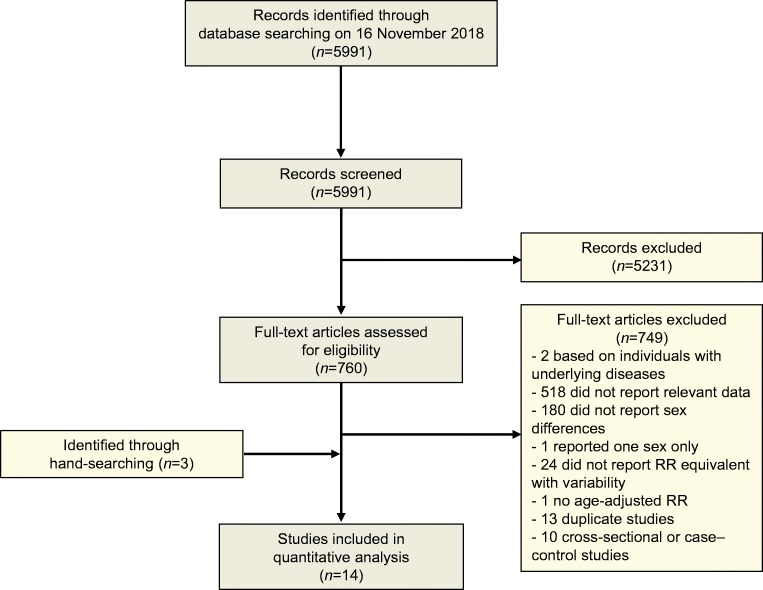
Flow chart of study selection

Of the 5991 articles identified by the systematic search, 760 articles qualified for full-text evaluation, and 14 articles provided summary data for sex differences in the association between diabetes and the risk of heart failure [5, 15–27] (Fig. 1).

The characteristics of all 14 studies included are shown in Table 1 and ESM Table 2. Data on type 1 diabetes and heart failure were available from two studies, involving two cohorts, including 3,284,123 individuals, and 95,129 events. Data on type 2 diabetes and heart failure were available from 13 studies, involving 47 cohorts, including 11,925,128 individuals, and 249,560 events, among which two studies, involving two cohorts, including 368,072 individuals, and 4584 events, reported age-adjusted RRs only. Nine studies provided data on absolute risks (ESM Table 3).

**Table 1:** Characteristics of the studies reporting on the association between diabetes and heart failure

Cohort	Country	Baseline years	Follow-up (years)	Study population	*N* (% women)	Age range (years)	*N* of diabetes (% women)	Type of diabetes	Ascertainment of diabetes	*N* of outcome (% women)	Fatal or non-fatal	Maximum adjustment available
APCSC [16] ^a^	Pool of 32 cohorts	1966–1999	7	Population-based, occupational settings	543,694 (36)	≥20	NA	Both	Self-reported, measured	496 (NA)	F	Age, SBP, BMI, cigarette smoking, regression dilution bias, study (stratified)
Policardo et al [23] ^b^	Italy	2008	5	Population-based	3,192,203 (NA)	≥16	152,954 (NA)	Both	Prescription, exemption from paying for diabetes, hospitalisation with diagnosis of diabetes	26,154 (55)	Both	Age, CCI, previous hospitalisations for other CVD
KPMCP [26]	US	1978–1984	9.5 median	Health maintenance organisation	64,877 (54)	≥40	NA	Both	Self-reported, measured	1330 (46)	Both	Age, race, education, HT, MI, frequent chest pain, TC, BMI, creatinine, uric acid, urine protein, LVH, smoking, alcohol ^c^
LRPP [25]	US, pool of 4 cohorts	1948, 1971, 1987–1989, 1967–1973	27.1, 20.3 (for index aged 45 and 55)	Population-based	19,249 (50), 23,915 (53) (for index aged 45 and 55)	30–62, 5–70, 45–64, ≥18	659 (46), 1792 (52) (for index aged 45 and 55)	Both	Measured, treatment	1677 (47), 2976 (52) (for index aged 45 and 55)	Both	Age, race, education, smoking status, HT, obesity
CHS [24]	US	1989–1990, 1992–1993	12.5 median	Population-based	4817 (61)	≥65	681 (53)	Both	Measured, treatment	1342 (57)	Both	Age, clinical site, education, smoking, alcohol consumption, BMI, physical activity
Swedish NDR (T1) [27]	Sweden	1998–2011	T1: 7.9, control: 8.3	Population-based (T1 was identified through NDR)	T1: 33,402 (45), control: 166,228 (45)	≥18	33,402 (45)	T1	NDR	2387 (39)	Both	Age, time-updated diabetes duration, birth in Sweden, educational level, baseline comorbidities
Swedish NDR (T2) [20]	Sweden	1998–2012	5.6 median	Population-based (T2 was identified through NDR)	T2: 266,305 (45), control: 1,323,504 (45)	T2: 62, control: 62 mean	266,305 (45)	T2	NDR	T2: 18,715 (46), control: 50,157 (45)	Both	Age, duration of diabetes, income, education, marital status, immigration status, stroke, acute MI, CHD, AF, renal dialysis or transplantation
Kaiser Permanente Georgia [17]	US	2000–2005	2.8	Health maintenance organisation	359,947 (53)	≥18	12,344 (49)	Both	Medical record, pharmacy claim	4001 (50)	Both	Age, HT, coronary artery disease, AF, valvular heart disease
NHANES I Epidemiologic Follow-up Study [15]	US	1971–1975	19	Population-based	13,643 (59)	25–74	521 (61)	Both	Self-reported	1382 (46)	Both	Age, race, education, physical activity, smoking, alcohol consumption, overweight, HT, valvular heart disease, CHD, BMI, SBP, TC, hypercholesterolaemia
Taiwan’s NHI system [19]	Taiwan	2000	T2: 7.8, control: 8.0	Population-based	T2: 34,291 (47), control: 34,291 (47)	60 mean	34,291 (47)	T2	Ambulatory care claims	8420 (51)	Both	Age, geographical area, urbanisation status, Hx of CHD, Hx of coronary revascularisation procedures, statins, β-blockers, diuretics
Saskatchewan Health databases [22]	Canada	1991–1996	5.2	Population-based	T2 11,881 (45), control: 552,765 (51)	≥30	11,881 (45)	T2	Prescription	2263 (46)	Both	Age
CALIBER programme [5]	UK	1998–2010	5.5 median	Primary care practices	1,921,260 (51)	≥30	34,198 (46)	T2	Medical record	13,938 (NA)	Both	Age, BMI, deprivation, HDL-C, TC, SBP, smoking, statin and antihypertensive drug prescriptions
Ballotari et al [21]	Italy	2011	3	Population-based	356,191 (51)	30–84	24,348 (44)	T2	Diabetes register	2321 (44)	Both	Age, foreign status ^d^
NHS Information Services Scotland [18]	UK	2004–2013	10	Population-based	T1: 18,240 (45), T2 136,042 (46), no diabetes 3,066,253 (54)	≥30	T1: 18,240 (45), T2: 136,042 (46)	T1, T2	Diabetes register	T1: 1313 (NA), T2: 22,959 (NA), no diabetes: 91,429 (NA)	Both	Age, socioeconomic status, calendar year ^e^

If endpoints were reported as incident, they were considered to include both fatal and non-fatal events, e.g. hospitalisation for heart failure

^a^*N* of total participants in APCSC was derived from overall participants. One out of 36 cohorts in APCSC (*n* = 12,203/543,694, 2.2%) consisted of male only

^b^*N* of total participants in Policardo et al was derived from overall participants (≥16 years old)

^c^RRs for controlled diabetes in participants aged <60 were extracted

^d^RRs were classified to be age-adjusted

^e^RRs for 2013 (aged ≥30 years) were extracted

AF, atrial fibrillation; APCSC, Asia Pacific Cohort Studies Collaboration; CALIBER, Cardiovascular disease research using LInked Bespoke studies and Electronic health Records; CCI, Charlson Comorbidity Index; CHS, Cardiovascular Health Study; COPD, chronic obstructive pulmonary disease; CVD, cardiovascular disease; F, fatal; HDL-C, HDL-cholesterol; HT, hypertension; Hx, history; KPMCP, Northern California Kaiser Permanente Medical Care Program; LRPP, Cardiovascular Disease Lifetime Risk Pooling Project (Framingham Heart, Framingham Offspring, Atherosclerosis Risk In Communities [ARIC], Chicago Heart Association Detection Project in Industry Study [CHA]); LVH, left ventricular hypertrophy; MI, myocardial infarction; NA, not available; NDR, National Diabetes Registry; NHANES I, First National Health and Nutrition Examination Survey; NHI, National Health Insurance; NHS, National Health Service; SBP, systolic BP; T1, type 1 diabetes; T2, type 2 diabetes; TC, total cholesterol

**Fig. 2:**
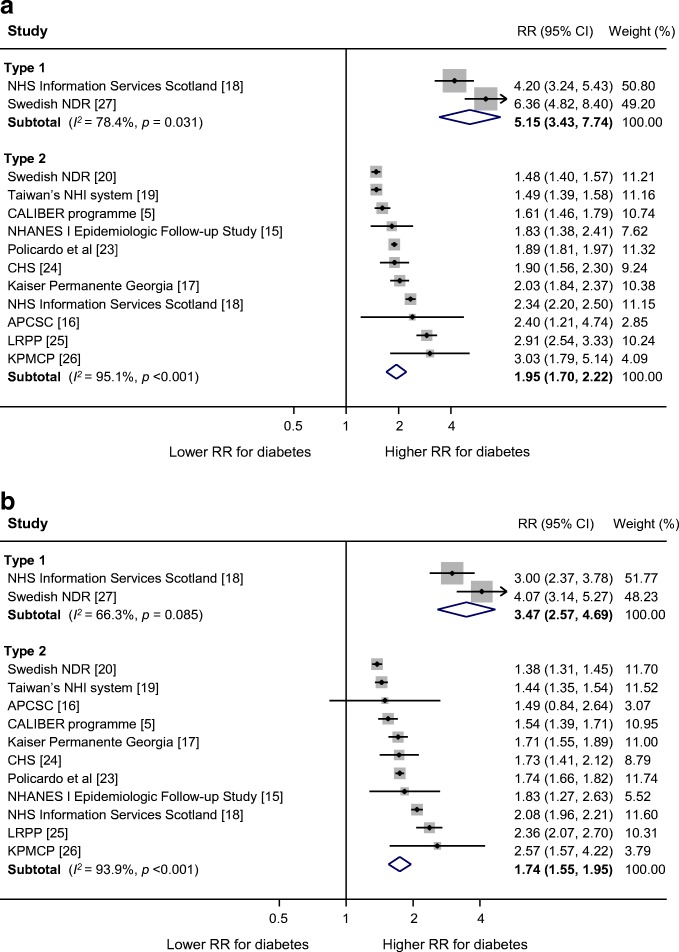
Multiple-adjusted RR for heart failure, comparing individuals with type 1 and type 2 diabetes with those without diabetes for (**a**) women and (**b**) men. APCSC, Asia Pacific Cohort Studies Collaboration; CALIBER, Cardiovascular disease research using LInked Bespoke studies and Electronic health Records; CHS, Cardiovascular Health Study; KPMCP, Northern California Kaiser Permanente Medical Care Program; LRPP, Cardiovascular Disease Lifetime Risk Pooling Project (Framingham Heart, Framingham Offspring, Atherosclerosis Risk In Communities [ARIC], Chicago Heart Association Detection Project in Industry Study [CHA]); NDR, National Diabetes Registry; NHANES I, First National Health and Nutrition Examination Survey; NHI, National Health Insurance; NHS, National Health Service

**Fig. 3:**
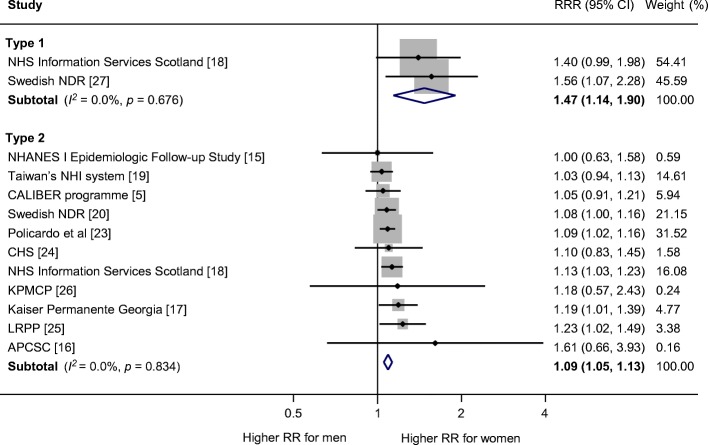
Multiple-adjusted women-to-men RRR for heart failure, comparing individuals with type 1 and type 2 diabetes with those without diabetes. APCSC, Asia Pacific Cohort Studies Collaboration; CALIBER, Cardiovascular disease research using LInked Bespoke studies and Electronic health Records; CHS, Cardiovascular Health Study; KPMCP, Northern California Kaiser Permanente Medical Care Program; LRPP, Cardiovascular Disease Lifetime Risk Pooling Project (Framingham Heart, Framingham Offspring, Atherosclerosis Risk In Communities [ARIC], Chicago Heart Association Detection Project in Industry Study [CHA]); NDR, National Diabetes Registry; NHANES I, First National Health and Nutrition Examination Survey; NHI, National Health Insurance; NHS, National Health Service

The multiple-adjusted pooled sex-specific RRs for heart failure associated with type 1 diabetes were 5.15 (95% CI 3.43, 7.74, *p* < 0.001) in women and 3.47 (2.57, 4.69, *p* < 0.001) in men (Fig. 2). The pooled multiple-adjusted RRR indicated a significantly greater excess risk for heart failure in women with type 1 diabetes compared with men. The women-to-men RRR was 1.47 (1.14, 1.90, *p* = 0.003 [Fig. 3]). The *I*^2^ statistics for heterogeneity between studies were 0.0%.

**Fig. 4:**
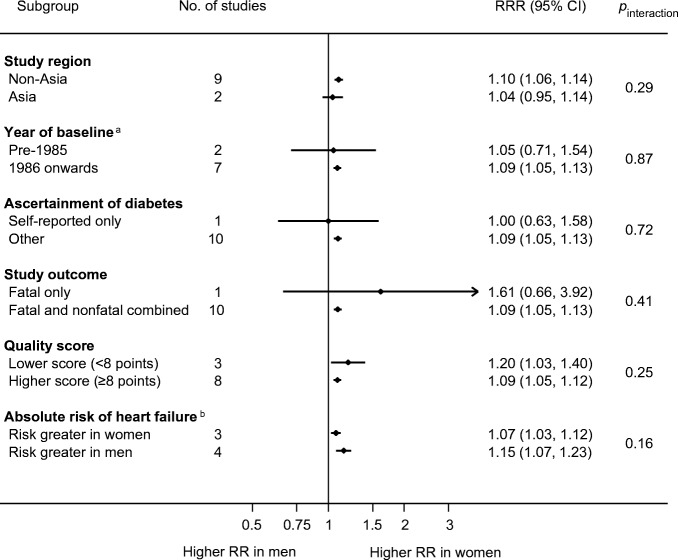
Subgroup analyses of multiple-adjusted women-to-men RRR for heart failure, comparing individuals with type 2 diabetes with those without. ^a^Year of baseline: two studies were excluded because baseline year bridged pre-1985 and 1986 onwards. ^b^Absolute risk of heart failure: absolute risk was derived using data from individuals with and without diabetes combined. Four studies were excluded because absolute risk was not available for both sexes

The sex-specific RRs for heart failure associated with type 2 diabetes was 1.95 (95% CI 1.70, 2.22, *p* < 0.001) in women and 1.74 (1.55, 1.95, *p* < 0.001) in men (Fig. 2), with the women-to-men RRR of 1.09 (1.05, 1.13), *p* < 0.001, *I*^2^ = 0.0% (Fig. 3). There was no evidence of publication bias for the association between type 2 diabetes and heart failure (Egger’s test *p* = 0.27, Begg’s test *p* = 0.31, ESM Fig. 1). In subgroup analyses, the pooled women-to-men multiple-adjusted RRR did not differ significantly by study region (*p* = 0.29), year of baseline study (*p* = 0.87), ascertainment of diabetes (*p* = 0.72), study outcome (*p* = 0.41), quality of study (*p* = 0.25), or absolute risk differences between men and women (*p* = 0.16) (Fig. 4).

For type 2 diabetes, five studies provided age-adjusted estimates. The pooled age-adjusted sex-specific RRs for heart failure associated with diabetes were 2.56 (95% CI 2.31, 2.84, *p* < 0.001) in women and 2.49 (2.04, 3.04, *p* < 0.001) in men. The pooled age-adjusted women-to-men RRR for heart failure was 1.00 (0.78, 1.27, *p* = 0.98). The *I*^2^ statistics for heterogeneity between studies was 87.6%, suggesting substantial heterogeneity.

In sensitivity analysis, restricted to studies which provided the sex-specific RRs for both multiple-adjusted and age-adjusted models, the pooled women-to-men RRR was 1.17 (95% CI 1.02, 1.35, *p* = 0.02) for multiple-adjusted analysis, and 1.19 (1.06, 1.34, *p* = 0.005) for age-adjusted analysis (ESM Fig. 2).

## Discussion

The present meta-analysis, of 47 cohorts including more than 12 million individuals, showed that both type 1 and type 2 diabetes were a stronger risk factor for heart failure in women than men. Type 1 diabetes was associated with a 47% greater excess risk of heart failure in women compared with men, and type 2 diabetes was associated with a 9% greater excess risk of heart failure in women than men. The sex difference in the association between type 2 diabetes and heart failure was consistent across a range of prespecified subgroups. These findings are in agreement with the previous evidence showing that diabetes has stronger associations with diabetic complications for women than men, and shed light on the importance of a routine sex-specific approach both in research and clinical practice in this field.

A previous meta-analysis reported that diabetes was associated with the risk of heart failure in both women and men [2]. However, this previous meta-analysis included studies consisting of women or men only, as well as studies among both women and men, and therefore could have introduced bias in quantifying sex differences. Further, sex-specific RRs were not reported for type 1 diabetes. The present meta-analysis includes additional two-sex studies that were not included previously, and provides evidence that both type 2 diabetes and type 1 diabetes are a risk factor for heart failure in both sexes, with significantly stronger associations in women than men. These findings suggest that healthcare providers and policy makers should be aware of this greater excess risk of heart failure, as well as other diabetic complications [8–11, 28, 29], in women than men.

In our analyses of type 2 diabetes, the women-to-men RRR was greater when multiple-adjusted RRs were pooled compared with when age-adjusted RRs were pooled (multiple-adjusted RRR 1.09 [95% CI 1.05, 1.13] vs age-adjusted RRR 1.00 [0.78, 1.27]). A significant degree of heterogeneity between studies was observed for age-adjusted analyses (*I*^2^ = 87.6%, *p* < 0.001), but not for multiple-adjusted analyses (*I*^2^ = 0.0%, *p* = 0.834). On the other hand, the sensitivity analysis including the studies that reported both multiple-adjusted and age-adjusted estimates provided almost similar results, indicating a greater excess risk of heart failure associated with diabetes in women than men. Therefore, we speculate that the difference observed between multiple-adjusted and age-adjusted analyses is likely due to chance differences between the studies included. Furthermore, we believe that multiple-adjusted estimates, which adjust for other major cardiovascular risk factors in addition to age, are more likely to represent true aetiology.

There are several potential explanations for the greater excess risk of heart failure associated with diabetes in women compared with men. First, the observed sex differences could be driven by there being a greater risk of CHD conferred by diabetes in women than men, because CHD is a major cause of heart failure in people with type 2 diabetes [1]. Our previous large-scale meta-analyses showed that diabetes conferred a 44% greater excess risk of incident CHD in women than men [8]. A significant sex difference was also observed in a meta-analysis which focused specifically on type 1 diabetes and CHD [28]. Sex differences in the management of diabetes could underpin these associations. Historically, women with diabetes had poorer glycaemic control than men with diabetes [30–34]. Second, in addition to CHD, undertreatment for women with diabetes could also contribute to the development of diabetic cardiomyopathy, a form of cardiac dysfunction that occurs independently of CHD and hypertension [35, 36], and could subsequently lead to a stronger association of diabetes with heart failure in women than men. Third, prolonged exposure to hyperglycaemia during the prediabetic state may also be involved. Women were reported to have 2 years longer duration of prediabetes than men [37]. Longer duration of prediabetes has been shown to be associated with left ventricular systolic and diastolic dysfunction [38]. Finally, it is also possible that sex differences in other cardiovascular risk factor profiles [30–33, 39–41] account for the greater excess risk of heart failure associated with diabetes in women compared with men. Deteriorations in major cardiovascular risk factor levels in individuals with diabetes compared with those without diabetes are reported to be greater in women than in men [9, 42, 43].

It might be also possible that the sex differences found in this study are a mathematical artefact caused by the relatively low absolute risk for heart failure in women compared with men. Suppose that the absolute risk difference following diabetes is the same in men as it is in women, then there would automatically be a larger RR among women compared with men. However, RRs, rather than absolute risk differences, are much more commonly reported in clinical studies, given their stability across different populations. No sex differences between women and men were found in our previous meta-analyses for risk factors and cardiovascular diseases [44, 45], which indicates that detection of a female disadvantage based on RRs is not inevitable.

Regarding type of diabetes, the excess risk of heart failure associated with diabetes was greater in type 1 diabetes than type 2 diabetes. The women-to-men RRR was 1.47 (95% CI 1.14, 1.90) for type 1 diabetes, and 1.09 (1.05, 1.13) for type 2 diabetes. The reason for this difference between type 1 and type 2 diabetes is unclear, but it may be partly explained by the above-mentioned sex differences in the association between diabetes and CHD. In our previous meta-analyses, type 1 diabetes showed a stronger sex difference in the association with incident CHD than type 2 diabetes, with women-to-men RRR of 2.54 (1.80, 3.60) for type 1 diabetes [28] and 1.44 (1.27, 1.63) for type 2 diabetes [8]. Future large-scale individual participant data meta-analysis and mechanistic studies might elucidate this difference.

The strengths of this meta-analysis are the large number of study participants and exclusion of studies which provided data for only one sex, which reduced the risk of both sampling and non-sampling error. This enabled us to provide robust evidence on the presence of sex differences in the risk of heart failure conferred by diabetes. Furthermore, the findings were consistent across a range of prespecified subgroups. Some limitations of this study should be mentioned. First, this meta-analysis was based on published data, with heterogeneity in study design, ascertainment of diabetes, definition of endpoint and extent of adjustment for confounding factors across studies. However, since we only included studies with results for both sexes, we minimised these issues by conducting within-study comparisons of the sexes. Second, there may be other unmeasured confounding factors in addition to those adjusted for in each study. Third, information on duration of diabetes, glycaemic control, glucose-lowering drugs or phenotype of heart failure was not available, and thus we cannot conduct detailed assessments regarding these factors. Analyses considering these factors would provide insight into potential explanation for the observed sex differences, and will be the subject of our future research. Fourth, the competing risk of premature death was not adjusted for in the present meta-analysis. Men with diabetes are at an increased risk of premature death compared with women with diabetes [46] (as indeed is the case in general populations), and therefore may be less likely to develop heart failure. This could partly explain the greater excess risk of heart failure following diagnosis of diabetes in women than men. Finally, we only found two studies of type 1 diabetes, which compromises the accuracy of our estimates in this regard. Additional studies are needed to address this issue.

In conclusion, the excess risk of heart failure following diagnosis of diabetes is significantly greater in women than men, highlighting the importance of intensive prevention and treatment of diabetes for women as well as men. Further research is required to understand the mechanisms underpinning the excess risk of heart failure conferred by diabetes (particularly type 1) in women and to reduce the burden associated with diabetes in both sexes.

## Electronic supplementary material

ESM (PDF 266 kb)

## Data Availability

The datasets generated during and/or analysed during the current study are available from the corresponding author on reasonable request.

## Abbreviation

RRR: Ratio of RR
