# Supplementary material for: Diabetes as a risk factor for heart failure in women and men: a systematic review and meta-analysis of 47 cohorts including 12 million individuals
Source: Diabetologia. 2019 Jul 18;62(9):1550–60. doi: 10.1007/s00125-019-4926-x (PMC6677875; doi:10.1007/s00125-019-4926-x)
Supplement: Supplementary file 1 — (PDF 266 kb) [file 125_2019_4926_MOESM1_ESM.pdf]

Electronic supplementary material

ESM text. Newcastle-Ottawa Quality assessment scale modified from reference 14

A study can be awarded a maximum of one point for each * within the Selection and Outcome categories. A maximum of two point can be given for Comparability.

Selection

S1) Representativeness of the exposed cohort

a) truly representative of the general population*

b) somewhat representative of the general population

c) selected group e.g. patient groups

d) no description of the derivation of the cohort

S2) Selection of the non exposed cohort

a) drawn from the same community as the exposed cohort*

b) drawn from a different source

c) no description of the derivation of the non exposed cohort

S3) Ascertainment of exposure

a) secure record*

b) secure record or written self report

c) written self report

d) no description

S4) Demonstration that outcome of interest was not present at start of study

a) yes*

b) no

Comparability

C1) Comparability of cohorts on the basis of the design or analysis

a) study controls for age*

b) study also controls for additional factors*

Outcome

O1) Assessment of outcome

a) independent blind assessment or confirmation by reference to secure records*

b) record linkage*

c) self report

d) no description

O2) Was follow-up long enough for outcomes to occur

a) yes (at least 3 years)*

b) no

O3) Adequacy of follow up of cohorts

a) complete follow up - all subjects accounted for*

b) subjects lost to follow up unlikely to introduce bias - > 90% follow up, or description provided of those lost*

c) follow up rate < 90% and no description of those lost

d) no statement

ESM Table 1. Search strategies

Pubmed (between Jan 1, 1966 and Nov 16, 2018, n = 5991, searched on Nov 16, 2018)

No	Search item
1	“Diabetes Mellitus” [MeSH Terms]
2	“Diabetes” [All Fields]
3	1 or 2
4	“Heart failure” [MeSH Terms]
5	“Heart failure” [All Fields]
6	“Cardiac failure” [All Fields]
7	“Cardiac insufficiency” [All Fields]
8	“Ventricular dysfunction” [MeSH Terms]
9	“Ventricular dysfunction” [All Fields]
10	4 or 5 or 6 or 7 or 8 or 9
11	“Men” [MeSH Terms]
12	“Male” [MeSH Terms]
13	“Men” [All Fields]
14	“Male” [All Fields]
15	11 or 12 or 13 or 14
16	“Women” [MeSH Terms]
17	“Female” [MeSH Terms]
18	“Women” [All Fields]
19	“Female” [All Fields]
20	16 or 17 or 18 or 19
21	“Cohort Studies” [MeSH Terms]
22	“Follow Up Studies” [MeSH Terms]
23	“Prospective Studies” [MeSH Terms]
24	“Longitudinal Studies” [MeSH Terms]
25	“Cohort” [All Fields]
26	“Follow-up” [All Fields]
27	“Prospective” [All Fields]
28	“Longitudinal” [All Fields]
29	21 or 22 or 23 or 24 or 25 or 26 or 27 or 28
30	3 and 10 and 15 and 20 and 29

ESM Table 2. Quality assessment of the included studies

Cohort	Scores								
	S1	S2	S3	S4	C1	O1	O2	O3	Sum
APCSC [16]	1	1	0	0	2	1	1	0	**6**
Policardo et al [23]	1	1	1	1	2	1	1	0	**8**
KPMCP [26]	1	1	0	1	2	1	1	0	**7**
LRPP [25]	1	1	1	1	2	1	1	0	**8**
CHS [24]	1	1	1	1	2	1	1	0	**8**
Swedish NDR (T1) [27]	1	1	1	1	2	1	1	0	**8**
Swedish NDR (T2) [20]	1	1	1	1	2	1	1	0	**8**
Kaiser Permanente Georgia [17]	1	1	1	1	2	1	0	0	**7**
NHANES I Epidemiologic follow-up study [15]	1	1	0	1	2	1	1	1	**8**
Taiwan's NHI system [19]	1	1	1	1	2	1	1	0	**8**
Saskatchewan Health databases [22]	1	1	1	1	1	1	1	0	**7**
CALIBER programme [5]	1	1	1	1	2	1	1	0	**8**
Ballotari et al [21]	1	1	1	0	1	1	0	0	**5**
NHS Information Services Scotland [18]	1	1	1	1	2	1	1	0	**8**

ESM Table 3. Absolute risks of heart failure in the included studies

Cohort	Men	Women	Difference (men - women)
APCSC [16]	na	na	
Policardo et al [23]	1.70	1.94	-0.24
KPMCP [26]	na	na	na
LRPP [25][a]	5.01	4.49	0.51
CHS [24]	28.46	20.85	7.62
Swedish NDR [20, 27][b]	7.11	7.16	-0.05
Kaiser Permanente Georgia [17]	4.24	3.68	0.56
NHANES I Epidemiologic follow-up study [15]	na	na	na
Taiwan's NHI system [19]	14.99	17.18	-2.19
Saskatchewan Health databases [22]	3.82	3.54	0.28
CALIBER programme [5]	na	na	na
Ballotari et al [21]	2.52	1.91	0.61
NHS Information Services Scotland (T2) [18] [c]	3.71	3.01	0.70

Incidence (or mortality) rates were described as per 1000 person-years.

na: not available.

[a] Weighted average of aged 45 and 55.

[b] Derived from reference [20].

[c] Subjects aged ≥30 years were included,.

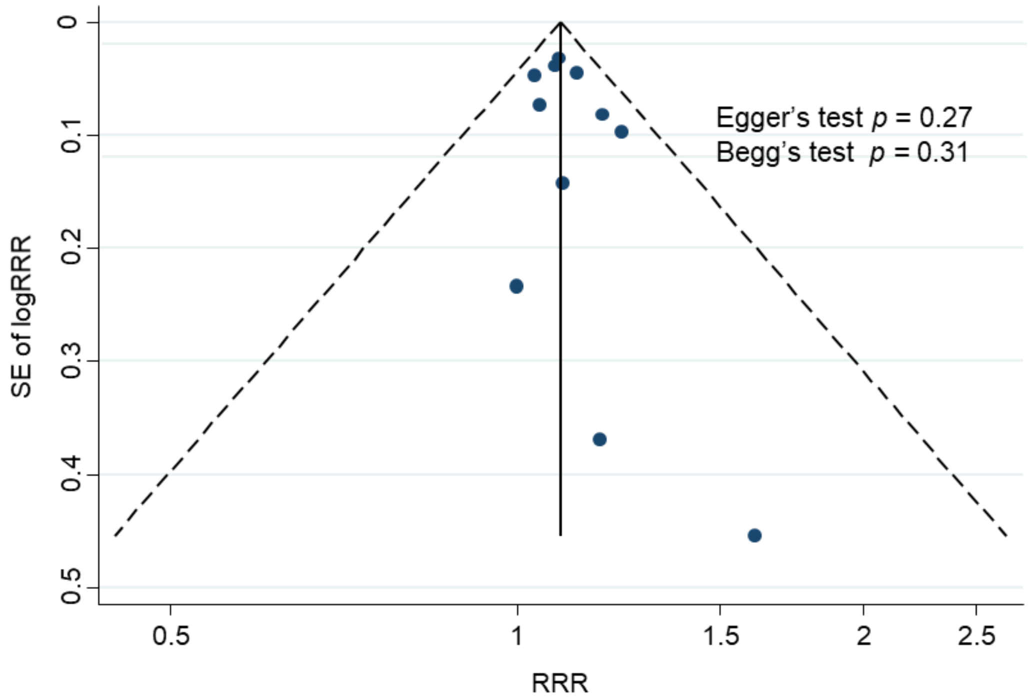

ESM Fig. 1. Funnel plot with pseudo 95% confidence limits for the data for type 2 diabetes in Fig. 3.

Abbreviations; RRR, ratio of relative risk; SE, standard error.

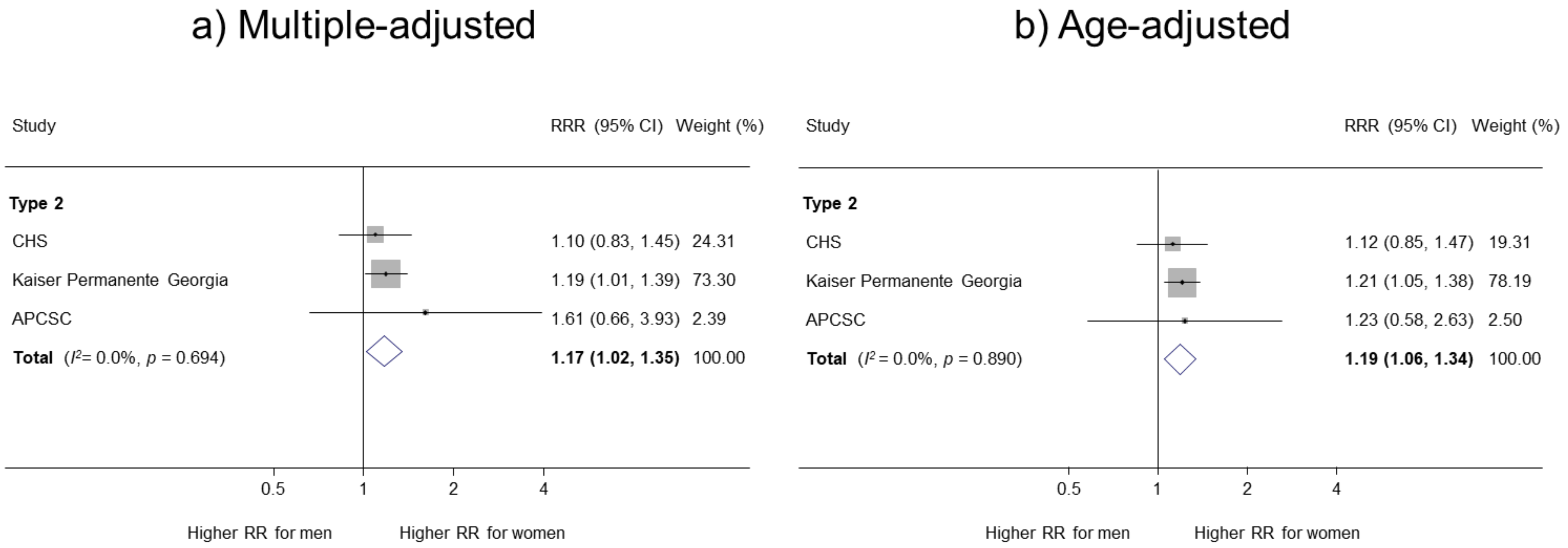

ESM Fig. 2. Women-to-men ratio of relative risk for heart failure, comparing individuals with diabetes with those without diabetes, which provided both multiple-adjusted and age-adjusted relative risk from the same study.

Abbreviations; RR; relative risk; RRR, ratio of relative risk; CI, confidence interval.
